# Volume Hologram Formation in SU-8 Photoresist

**DOI:** 10.3390/polym9060198

**Published:** 2017-05-30

**Authors:** Tina Sabel

**Affiliations:** Department of Chemistry, Technische Universität Berlin, Strasse des 17. Juni 135, Berlin 10623, Germany; tina@physik.tu-berlin.de

**Keywords:** photosensitive polymers, volume hologram formation, photo curing, diffraction, phase gratings, absorption gratings

## Abstract

In order to further understand the mechanism of volume hologram formation in photosensitive polymers, light-induced material response is analyzed in commonly used epoxy-based negative photoresist Epon SU-8. For this purpose, time-resolved investigation of volume holographic grating growth is performed in the SU-8 based host–guest system and in the pure SU-8 material, respectively. The comparison of grating growth curves from doped and undoped system allows us to draw conclusions on the impact of individual components on the grating formation process. The successive formation of transient absorption as well as phase gratings in SU-8 is observed. Influence of exposure duration and UV flood cure on the grating growth are investigated. Observed volume holographic grating formation in SU-8 can be explained based on the generation and subsequent diffusion of photoacid as well as time-delayed polymerization of exposed and unexposed areas.

## 1. Introduction

Volume holography represents a very interesting field of application for photo-responsive polymers. The mechanism of volume hologram formation in photosensitive polymers, a complex process where several components are involved, is attributed to the interplay of polymerization and diffusion, induced by a spatially modulated holographic exposure [1]. Advanced analytical methods and sophisticated models are required for further understanding of volume holographic grating formation in photo-responsive polymers [2,3,4]. A particularly useful tool to draw conclusions on the mechanism of volume hologram formation is to study the light-induced material response.

Many factors influence how a photosensitive material responds to light during a holographic exposure. The material response depends on intrinsic material parameters, such as material composition or viscosity as well as on recording parameters, such as exposure duration and recording intensity [5,6].

There are different approaches for studying the material response. The subject and starting point of the investigation is the optical functionality of the volume holographic grating, represented by diffraction efficiency and Bragg selectivity in terms of angular or wavelength selectivity [2]. Those investigations may be based on angular resolved analysis [5], spatially resolved analysis [6], and/or time resolved analysis [7,8], respectively. It is also possible to draw conclusions on the material response based on thorough examination of the grating parameters. In this case, imaging techniques may also be applied [9].

Photosensitive polymers have been used as holographic media since 1969 [10]. Polymers combine many advantages, namely low cost, ease of fabrication and high flexibility. They fulfill the requirements for volume holographic recording with no need for solvent processing, good dimensional stability, variable thickness, high energetic sensitivity, large dynamic range, and sharp angular selectivity [11].

Grating formation in photosensitive polymers occurs primarily as a consequence of photopolymerization and mass transport processes [12]. A light pattern is projected into the photosensitive medium, inducing local polymerization proportional to the light intensity. As a result, a chemical gradient is induced, followed by monomer diffusion and subsequent polymerization. The final grating is formed as a periodic modulation of optical properties, according to the recording light pattern [2]. The corresponding optical functionality of the volume holographic grating can be assigned to a modulated refractive index (phase gratings) or absorption (absorption gratings), as well as mixed forms [13].

In order to study the physico-chemical material transformations, responsible for grating formation in photosensitive polymers, it appears appropriate to simplify the material composition as far as possible. Host–guest systems particularly provide the possibility to compare the doped host–guest system with the undoped host system.

A new organic photosensitive material for volume holographic recording, based on an epoxy host–guest system, has recently been introduced [5]. Here, grating growth curves revealed a transition of the refractive index contrast [7].

**Figure 1:**
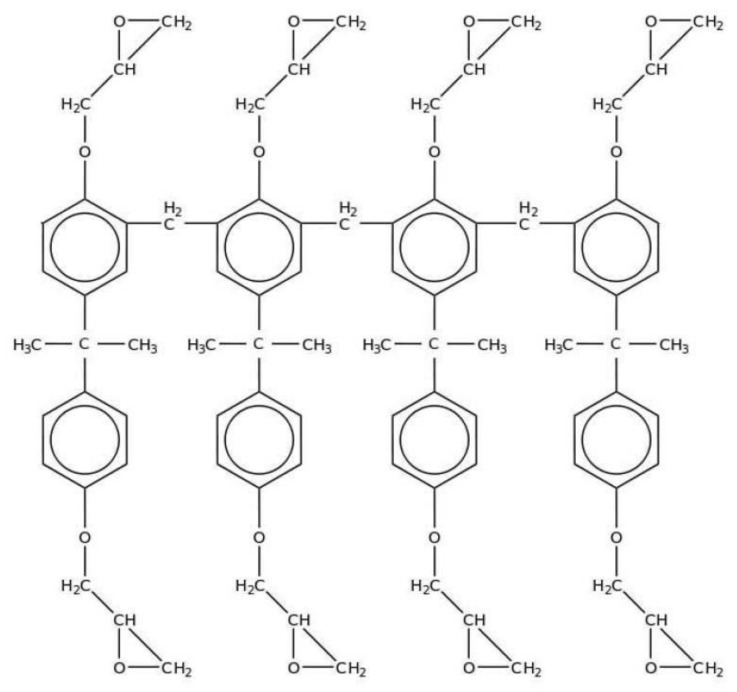
Chemical structure of a single SU-8 molecule.

The corresponding host system provides a simple system to study the material kinetics: the negative photoresist SU-8 consists of epoxy resin, organic solvent, and photoinitiator [14]. A single SU-8 molecule is shown in Figure 1. The epoxy group is aromatically bound. The high functionality of the epoxy group provides high sensitivity to the photoresist [15]. The corresponding mechanism of polymerization is a cationic ring-opening polymerization (CROP).

The Epon resin features an average of eight functional groups per repeating unit (see Figure 1) [16]. This results in a high degree of cross-linking after curing. As a consequence, the final sample possesses excellent chemical resistance, high temperature resistance, and high dimensional stability [17]. Apart from that, a high cross-linking density can also be accompanied by lower flexibility and higher rigidity. In view of the proposed application as volume holographic system, this can also be a drawback. Here, grating formation occurs as an all-optical process, with no need for post-exposure chemical treatment. Therefore, high diffusion rates are required in order to achieve a high refractive index contrast [18].

As a result, the excellent applicability of SU-8 for various lithography techniques notwithstanding [19], SU-8 photoresist without diffusing component appears to not be qualified for use in volume holography [5]. Nevertheless, it is shown here that volume holographic gratings can be recorded in SU-8. Based on these results, it is possible to compare the respective material response of doped and undoped system to draw conclusions on the impact of individual components and on the interaction of host and guest components throughout the hologram formation process.

## 2. Materials and Methods

### 2.1. Sample Preparation

Free-surface, ultraviolet curable epoxy (Epon SU-8) samples are prepared by micro resist technology GmbH. The host–guest material composition is based on epoxy oligomer. Both, host and guest molecules feature epoxy functional groups, with the corresponding CROP mechanism. The oligomer host system features an aromatic bond of the epoxy group. In case of the monomer guest, the epoxy group is bound aliphatically. Tack-free films are obtained up to a guest content of 17% by weight. A sensitized photoacid generator (PAG) is used to induce crosslinking by cationic polymerization at 405 nm [5]. A schematic illustration for the composition of doped and undoped system is provided in the conclusion.

Spin coating of SU-8 on glass substrates with rotation speed of 800 min^−1^ results in layers with thickness of 200 μm. Subsequent pre-exposure bake is carried out on a hotplate (80 °C) for 30 min, driving out remaining solvent in order to receive a tack-free film.

For more details on the host–guest system, in terms of composition as well as performance, such as energetic sensitivity and angular selectivity, and on the recording setup, see [5].

### 2.2. Holographic Exposure

Investigations are based on one-dimensional, plane-wave, transmission type volume holographic gratings. Symmetric recording geometry results in unslanted gratings with a periodicity of Λ ≈ 2 μm.

Holographic exposure is performed by two freely propagating, s-polarized recording beams with 405 nm wavelength and 2 mm beam diameter.

After completion of holographic grating formation, samples are fixed by UV flood cure with a dose of 350 mJ/cm^2^. Remaining photoinitiator is used up during this curing step, resulting in a sample which is no longer light-sensitive. No postbake, hardbake, or any additional developing was applied.

### 2.3. Real-Time Observation of Holographic Grating Growth

Grating growth curves are obtained by monitoring the time evolution of the diffracted part of a probe beam from the very start of exposure. Such in-situ techniques enable real-time, non-disturbing observation of the grating formation process [2,7].

To ensure non-disturbing observation, the in situ probe wavelength was chosen outside of the absorption spectrum of the photosensitizer dye. A fiber-guided 633 nm HeNe laser was used in combination with an adjustable collimator. This allows probing with a slightly focused beam to steadily ensure a stable on-Bragg condition. A position sensitive device (PSD) was used to detect the diffracted light. The PSD provides time-resolved information on the diffraction efficiency.

## 3. Results and Discussion

### 3.1. Time Response

**Figure 2:**
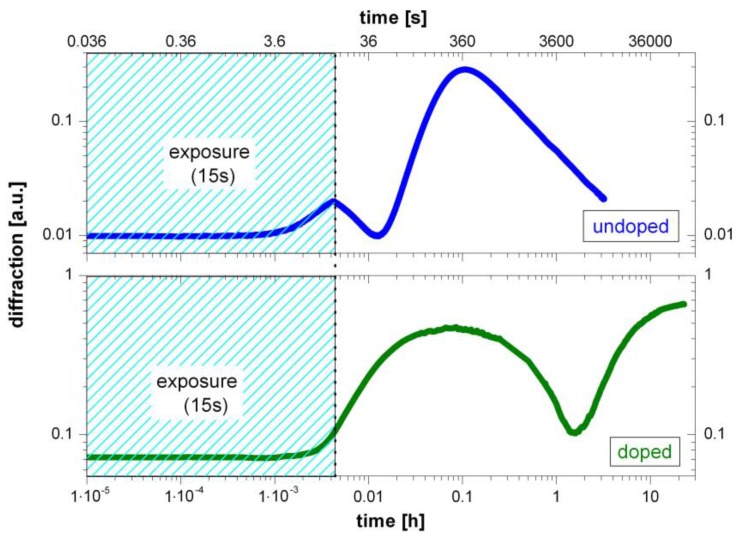
Volume holographic grating growth: Comparison of growth curves from the undoped (**top**) and doped system (**bottom**) on double logarithmic scales. Exposure duration was 15 s in each case.

Volume holographic grating growth curves from the doped and undoped systems are shown in Figure 2. Holograms were recorded under equal conditions. Exposure duration was 15 s in both cases. As expected, the two growth curves differ significantly from each other. In the case of the doped system, the characteristic two-step growth can be attributed to a transition of the refractive index contrast from positive to negative values, as a result of competing effects, taking place on overlapping time scales [7]. The underlying mechanism, responsible for grating formation, is a polymerization-induced change of the refractive index. However, it is unexpected to also find a two-step grating growth in case of the undoped system.

Apart from the unexpected fact that a two-step grating growth was found for both systems, comparison of the final states appears to be in line with the expectations. As can be seen from Figure 2, the grating in the doped system is going into saturation, while the hologram degenerates in case of the undoped system. The holographic grating in SU-8 is not stable because without diffusing component the undoped system is heading for a completely polymerized end state. Although the holographic exposure initiates local polymerization only in the areas corresponding to high intensity of exposure, over time the dark areas also polymerize. The growth curve maximum corresponds to the highest contrast between dark and bright areas. This state is reached as soon as almost all functional groups are cross-linked in the exposed area. In the case of growth curves shown here, this state is reached after approximately 6 minutes. Subsequently, diffusion of generated photoacid may account for polymerization of the dark areas [20]. If no mass transport has taken place by means of component diffusion, the polymerization-induced contrast between dark and bright areas disappears. As a consequence, the hologram is finally depleted.

**Figure 3:**
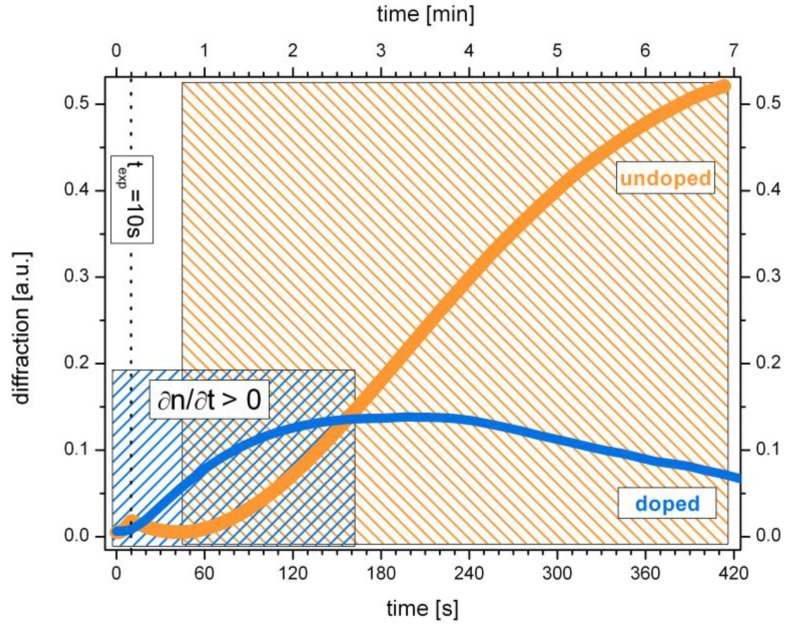
Direct comparison of the first growth step on a real time scale. Exposure duration was 10 s for both the undoped (orange) and doped system (blue), respectively. The shaded areas correspond to the time frames with positive change of the refractive index (∂*n*/∂*t* > 0).

The significant differences in time response of the doped and undoped system, already observed in view of Figure 2, become even more apparent on a real time scale, see Figure 3. The corresponding holograms are generated under equal conditions. Exposure duration was 10 s in each case.

Although both systems possess the particular characteristic two-step grating growth, it can be seen from Figure 3 that the first growth steps in the doped and undoped system differ significantly from each other. These differences concern the time response as well as the shape of the growth curve, respectively. The first growth step in case of the undoped SU-8 is much shorter and less pronounced compared to the first growth step in the doped system. Furthermore, results from Figure 2 and Figure 3 show that, in case of the undoped SU-8, the first growth step stops abruptly with the end of the exposure. This suggests a direct correlation of the first growth step with the exposure duration, which will be investigated more closely in the next section.

### 3.2. Impact of the Exposure Duration

**Figure 4:**
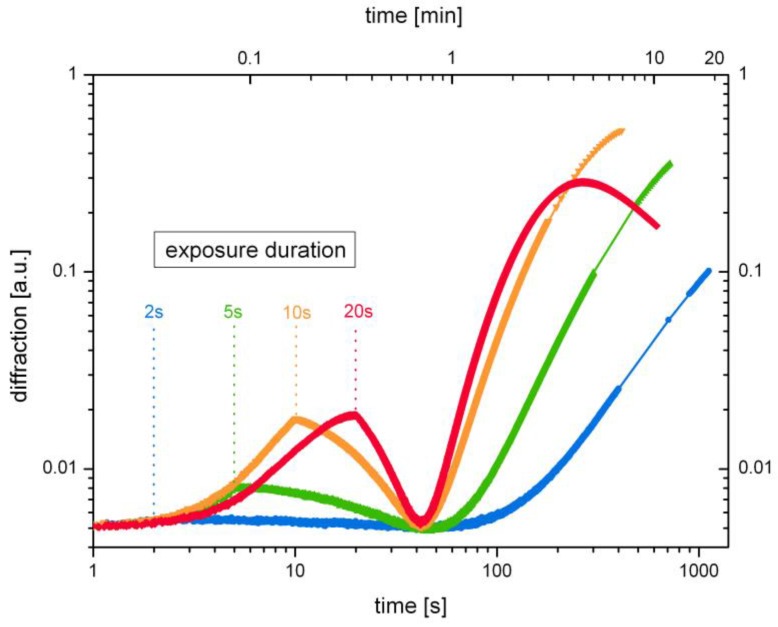
Grating growth curves in the undoped SU-8 on a double logarithmic scale for various exposure durations.

To draw conclusions on the material response and to understand the underlying mechanisms, it is useful to study the grating growth under variation of important influential factors. Figure 4 shows the influence of the exposure duration on the grating growth curves in undoped SU-8. The impact of the exposure duration becomes particularly apparent on a double logarithmic scale.

Results shown in Figure 4 confirm the direct correlation of the first growth step with the exposure. In fact, the SU-8 grating growth curves show a characteristic performance, depending on the duration of the exposure. The first growth step can directly be assigned to the exposure process. Here, the diffraction efficiency rises as long as the hologram is exposed. This growth abruptly stops with the end of the exposure, followed by a depletion of diffraction. Subsequently, a long-term effect is observed. This second growth step can be attributed to a positive change of the refractive index, related to polymerization, which is in line with the expectations, as explained above. In the course of the second growth step, diffraction efficiency rises up to the saturation, corresponding to the growth curve maximum and to the highest contrast between dark and bright areas. This is followed by self-destruction of the hologram (see Figure 2 and red curve in Figure 4), corresponding to polymerization of the dark areas, as described in Section 3.1.

The influence of the exposure duration becomes apparent in view of Figure 4. The longer the exposure lasts, the stronger the diffraction efficiency rises during the second growth step. With respect to the grating formation mechanisms this can be considered a result of photoacid generation: SU-8 is polymerized by photoacid generation. The longer the exposure lasts, the more photoacid is produced. As a consequence, a faster material response can be observed with regard to the second growth step. Whereas the earlier start of saturation and hologram degradation, particularly apparent in view of the last growth curve in Figure 4 (exposure duration 20 s), may also be a result of pre-exposure influence (the four corresponding holograms shown in Figure 4 are subsequently generated in one sample, starting with the shortest exposure (2 s), towards the longest exposure (20 s)).

### 3.3. Explanation Approach for the First Growth Step

A possible explanation for the first growth step in the undoped SU-8 is the formation of an absorption grating. Contributions of absorption and phase gratings to the total diffraction efficiency of volume holographic gratings have already been subject of research on glass-like polymer recording materials [21]. It has also been observed in case of nanocomposite materials, in conjunction with a multicomponent diffusion process [22].

**Figure 5:**
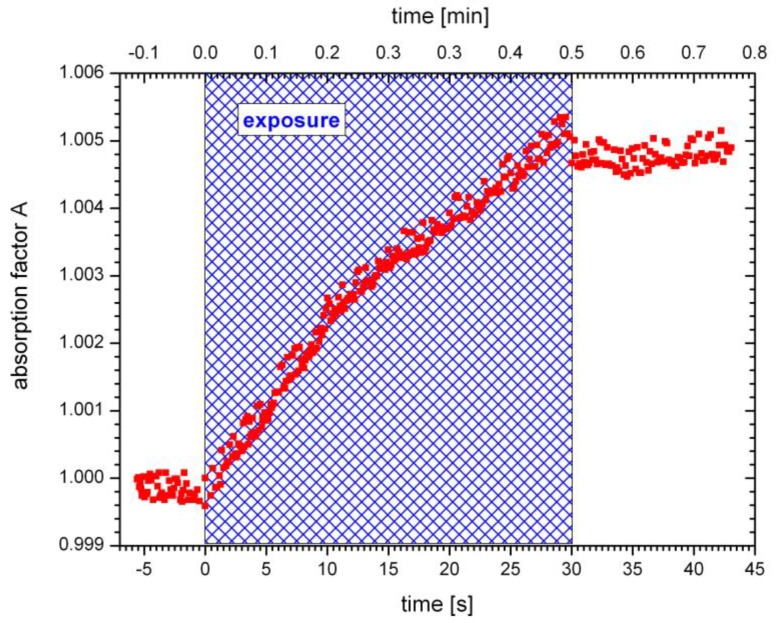
Measured absorption for the undoped SU-8 during single beam exposure. The absorption factor increases during exposure. Exposure duration was 30 s (shaded area).

To corroborate this hypothesis of an absorption grating formation, causing the first growth step in the case of the undoped SU-8, the absorption factor of the material was measured. For this purpose, transmitted light was detected while the sample was exposed with a single beam. Single beam exposure results in cross-linking of the sample across the exposure beam diameter. Figure 5 shows corresponding results.

Figure 5 corroborates the theory of absorption grating formation by showing that the exposure causes an increase of the absorption. During the exposure, lasting 30 s, the absorption factor rises significantly.

Photoacid is generated during exposure. As a consequence and with regard to the underlying mechanisms, the absorption grating may be assigned to the presence of photoacid. Optical inhomogeneities, responsible for the increasing absorption and the absorption grating formation may be caused by the photoacid molecules. Usually, the effect of photoacid diffusion is neglected because the migration of photoacid molecules is limited by the low flexibility of the resist film and rapid polymerization of the exposed regions [20]. However, the diffusion coefficient of the acid molecule strongly depends on the concentration of remaining solvent in the resist film [20]. Finally, the absorption grating is destroyed when the photoacid is consumed in the course of polymerization.

A possible explanation for the fact that no absorption grating has been observed in case of the doped host–guest system might be represented by the different reactivity of the functional groups in the doped and undoped systems. In fact, a different time response would be expected in view of the different reactivity of the aliphatically bound epoxy group compared to the reactivity of the aromatically bound functional group in the undoped system. It appears reasonable to expect a lower reactivity in case of the aromatically bound functional group. This corresponds to a faster material response of the doped system, compared to the undoped system. As a consequence, generated photoacid is rapidly consumed in case of the doped system and no absorption grating is formed.

### 3.4. Impact of Flood Exposure

**Figure 6:**
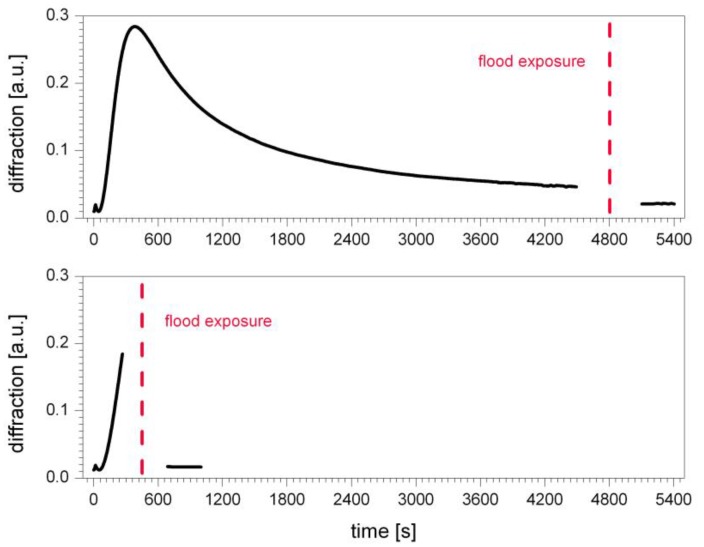
Impact of UV flood exposure on volume holographic grating growth in SU-8.: flood exposure results in an abrupt stop of the grating growth and erasure of the hologram, regardless of the implementation time. Fixation is performed during saturation state (**top**) and during maximum diffraction (**bottom**). In both cases the flood exposure results in destruction of the grating.

To further corroborate the assumptions made above, the impact of UV flood exposure on the grating growth has been evaluated. Results are shown in Figure 6.

It can be seen from Figure 6 that the flood exposure results in an abrupt stop of the grating growth. It does not matter when the flood exposure is started or to which extend the grating growth has proceeded. In any case, fixation results in immediate erasure of the hologram. Again, this is in line with the expectations: UV flood exposure initiates polymerization throughout the sample, resulting in complete cross-linking of the sample. As explained above, without component diffusion this results in fading of contrast between dark and bright areas.

However, a non-zero diffraction efficiency after flood exposure can be observed. The value of this non-zero diffraction efficiency after flood exposure corresponds to the maximum diffraction efficiency of the first growth step, which amounts 7% of the total maximum diffraction efficiency (see Figure 6). In terms of real diffraction efficiency, defined as the ratio of the input readout power to the diffracted power, the non-zero diffraction efficiency after flood exposure amounts to approximately 1–2%. The residual diffraction properties can be explained by the formation of a permanent phase grating. This indicates a possible diffusion of molecules with lower molecular weight. This appears reasonable based on the fact that Epon SU-8 features a certain molecular weight distribution and includes also smaller molecules, namely SU-1, SU-2, SU-4, and SU-6 [14].

## 4. Conclusions

It was shown that the undoped SU-8 photoresist is capable of forming volume holograms. A two-step grating growth has been observed. Table 1 gives an overview on how first and second growth step are assigned to different types of gratings in case of the doped and undoped system, respectively.

**Figure 7:**
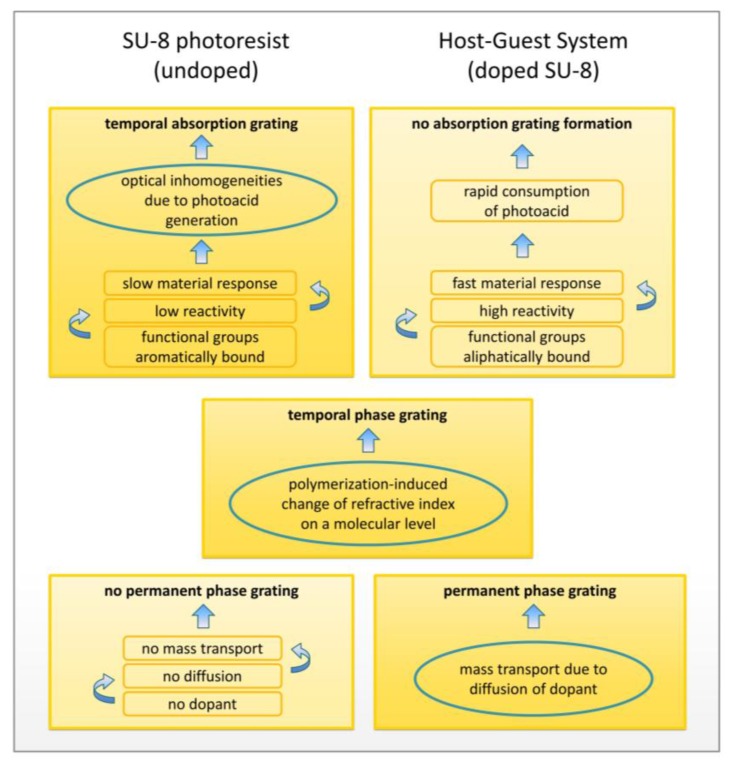
Overview of results and assumptions: Types of volume holographic gratings, found in doped and undoped SU-8, are contrasted. Results can be explained by specified attributes and underlying mechanisms, assumedly causing the presence or absence of respective gratings.

The comparison of volume holographic grating growth curves in doped and undoped SU-8, as well as investigations on the influence of exposure duration and flood exposure, have led to the following assumptions (Figure 7 gives an overview).

In SU-8, the first growth step appears to be a consequence of the formation of an absorption grating, assumedly due to photoacid generation, while the second growth step can be attributed to a positive change of the refractive index in the course of polymerization, resulting in a phase grating. Both absorption, as well as phase grating, are not stable in time. The absorption grating is fading away when photoacid is consumed in the course of polymerization. In the case of the phase grating, self-destruction of the hologram corresponds to polymerization of the unexposed areas as a consequence of photoacid diffusion. However, a non-zero permanent diffraction efficiency was found, potentially caused by diffusion of smaller molecules, such as SU-1, SU-2, and/or SU-4.

**Figure 8:**
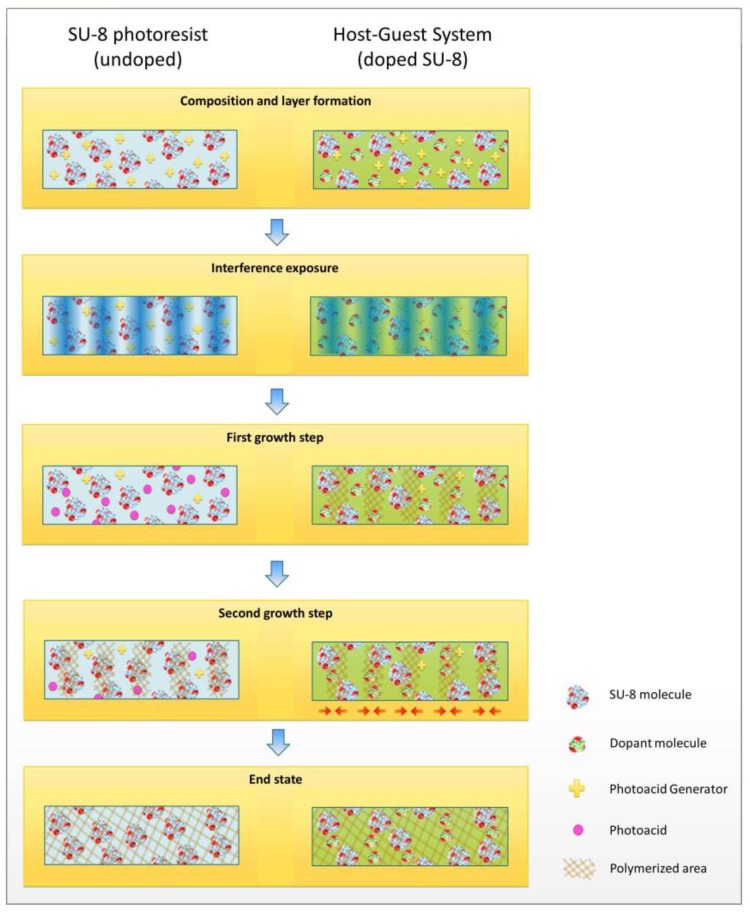
Graphical representation of grating growth in the doped and undoped system, respectively. The material composition is schematically displayed in the top line. Below, holographic interference exposure is shown. The first growth step in the undoped system is attributed to photoacid generation. Local polymerization causes the first growth in case of the doped system and the second growth step in case of the undoped system. The second growth step in the doped system can be attributed to monomer diffusion. The final state is a completely polymerized system in both cases, while the diffusion of dopant molecules has induced a permanent grating in case of the doped system.

To contrast the different grating formation mechanisms in the doped and undoped system, Figure 8 shows a graphical representation of the compositions and transient as well as permanent phenomena of polymerization and diffusion causing the different growth steps.

Altogether, results merge in a consistent overall picture on volume holographic grating growth in SU-8 material, on the underlying mechanisms of polymerization and diffusion, as well as on the impact of individual components.

## Figures and Tables

**Table 1:** Overview on types of volume holographic gratings, assumedly causing first and second growth step in case of doped and undoped SU-8, respectively.

Grating Growth	SU-8 Photoresist (Undoped)	Host–Guest System (Doped SU-8)
First growth step	Temporal absorption grating	Temporal phase grating
Second growth step	Temporal phase grating	Permanent phase grating
